# An unusual case: Adult T-cell leukemia/lymphoma in pediatric patient with human T-lymphotropic virus 1 infection. Clinical implications and therapeutic challenges

**DOI:** 10.1016/j.jdcr.2025.05.027

**Published:** 2025-06-18

**Authors:** Eine Yesid Benavides - Tulcan, Melissa Gutierrez - Gomez, Ricardo Augusto Rueda - Plata

**Affiliations:** Department of Internal Medicine, Dermatology Section, Health Faculty, Universidad del Valle, Cali, Colombia

**Keywords:** adult T cell leukemia/lymphoma, HTLV infection, HTLV virus, oncology, pediatric dermatology

## Introduction

Human T-lymphotropic viruses (HTLVs) are retroviruses classified into 2 main groups: HTLV-1 and HTLV-2; these viruses infect CD4+ and CD8+ T lymphocytes, respectively.^1^ Most patients infected with HTLV-1 remain asymptomatic; however, a small percentage develop adult T-cell leukemia/lymphoma (ATLL).^2^ Approximately 6% to 7% of male and 2% to 3% of female HTLV-1 carriers develop ATLL after a latency period of 30-50 years postinfection. ATLL is considered almost exclusively adult-onset and is extremely rare in the pediatric population.^3^^,^^4^

## Case

**Fig 1:**
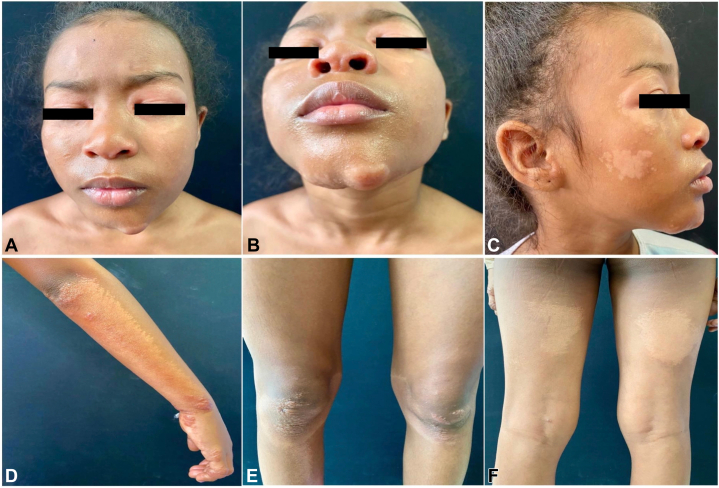
**A-F,** Adult T-cell leukemia/lymphoma. Papules, plaques, and infiltrated nodules accompanied by hypopigmented macules on the face and upper and lower extremities.

We report the case of an 8-year-old girl from the Colombian Pacific region, with no prior medical history, who presented with papules, plaques, and infiltrated nodules appearing since her first year of life. These were accompanied by hypopigmented macules on her face, upper and lower extremities, and associated lymphadenopathy (Fig 1).

**Fig 2:**
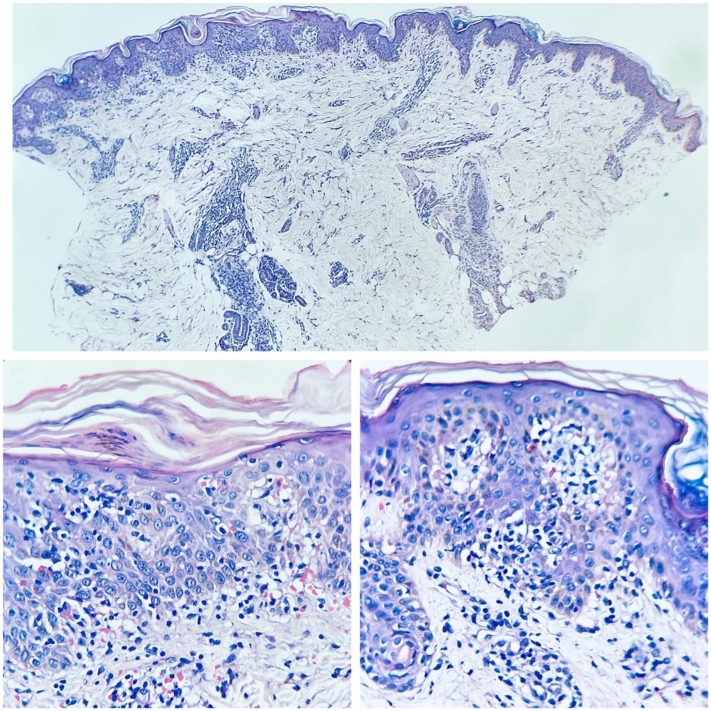
H&E. Adult T-cell leukemia/lymphoma. Nodular and interstitial lymphohistiocytic proliferation. *H&E*, Hematoxylin and eosin.

Suspecting a lymphoproliferative syndrome, a skin biopsy and complementary studies were performed. Histopathology (hematoxylin and eosin) revealed a dense lymphohistiocytic infiltrate in the dermis, arranged around the dermal plexuses, infiltrating cutaneous appendages, nerve filaments, and eccrine units (Fig 2). Immunohistochemistry was positive for CD3, CD4, CD5, CD7, CD8, and CD43. Blood tests showed leukemoid reaction and atypical lymphocytes with cleaved nuclei in the peripheral smear.

Given the patient’s geographic origin, serological tests for HTLV-1 and HTLV-2 were conducted, yielding positive results. Peripheral blood flow cytometry revealed an abnormal population comprising 39.5% of total events, corresponding to mature CD4+ T cells with an aberrant immunophenotype: CD45+, CD2+, CD3+, CD4+, weak CD5+, CD25+ (95%), partial CD7 expression, and negative for CD8. Bone marrow flow cytometry demonstrated a similar abnormal T-cell population representing 48.85% of total events, with an inverted CD4/CD8 ratio (15:91) and aberrant expression of CD45+, CD2+, CD3+, CD4+, CD5+, CD25+ (98%), CD7+/−, and weak CD8+/−, consistent with mature T-cell neoplasia. The clinical presentation, HTLV-1/HTLV-2 seropositivity, consistent CD25 expression, partial CD7 loss, bone marrow involvement, and aggressive clinical course confirmed acute-phase ATLL. The patient received 3 cycles of the LSG-15 chemotherapy protocol (vincristine, cyclophosphamide, doxorubicin and prednisone [VCAP]; doxorubicin, ranimustine, and prednisone [AMP]; vindesine, etoposide, carboplatin, and prednisone [VECP]); however, no clinical improvement was observed. Immunophenotyping of both bone marrow and cerebrospinal fluid revealed infiltration by aberrant lymphocytes, indicating relapse involving both the bone marrow and central nervous system. The patient was not considered a candidate for hematopoietic stem cell transplantation due to both parents testing positive for active HTLV-1 infection, and being an only child, no suitable sibling donors were available. Given these limitations, salvage chemotherapy was offered, but the patient’s parents ultimately declined curative treatment. She is currently undergoing palliative care, with a focus on symptom management.

## Discussion

HTLV-1 is endemic in regions such as Japan, Sub-Saharan Africa, and South America. In Colombia, the estimated prevalence based on blood donor seroprevalence studies ranges from 0.1% to 2.6%.^5^ Transmission occurs primarily through sexual, parenteral, and vertical routes.^1^ HTLV-1 is mainly associated with ATLL and tropical spastic paraparesis/HTLV-1–associated myelopathy, in addition to other conditions such as arthropathies, uveitis, and dermatological disorders, including infective dermatitis and folliculitis decalvans.^1^^,^^2^

The risk of progression to ATLL in infected patients ranges between 2% and 4%. The disease typically develops after a long latency period, with an average presentation age of approximately 60 years, making pediatric cases exceptionally rare.^1^

ATLL is classified in 4 variants: acute, lymphomatous, chronic, and indolent. In children, the acute variant is the most common and usually manifests with skin lesions, lymphadenopathy, hepatomegaly, and splenomegaly.^1^, ^2^, ^3^

Histopathologically, perivascular, nodular, and diffuse infiltration patterns can be observed, along with epidermotropism and Pautrier microabscesses. Although these findings are helpful, they lack diagnostic specificity, requiring high clinical suspicion.^3^ Thus, the diagnosis of ATLL is confirmed by HTLV-1 infection in a patient with mature T-cell neoplasia proven by histology and/or cytology, as in this case.^6^, ^7^, ^8^

The prognosis of ATLL is poor, with high relapse rates and limited survival. Treatment is not standardized and includes options such as interferon-alpha, zidovudine, combined chemotherapy, allogeneic hematopoietic stem cell transplantation, and new therapeutic agents.^5^^,^^8^ In this case, the patient did not respond to initial treatment, and other rescue options are currently under evaluation.

## Conclusions

The term ATLL is misleading, as it can also occur in children, as demonstrated in this case. ATLL is an aggressive T-cell neoplasm caused by chronic HTLV-1 infection. It is challenging to diagnose, has a poor prognosis, and represents a public health issue in HTLV-1 endemic regions of Latin America. Education about transmission pathways, early serological detection, elective cesarean sections, and breastfeeding suppression in seropositive women are key measures to mitigate the impact of this disease.

### Declaration of generative AI and AI-assisted technologies in the writing process

During the preparation of this work the authors used Grammarly Premium for Mac 2024 and Chat GPT-3.5 by Open AI in order to improve language and readability. After using these tools, the authors reviewed and edited the content as needed and take full responsibility for the content of the publication.

## Conflicts of interest

None disclosed.
